# Powder Diffraction: Least-Squares and Beyond

**DOI:** 10.6028/jres.109.008

**Published:** 2004-02-01

**Authors:** W. I. F. David

**Affiliations:** ISIS Facility, Rutherford Appleton Laboratory, Chilton, Oxon, OX11 0QX, U.K.

**Keywords:** least squares analysis, powder diffraction, Rietveld analysis

## Abstract

This paper addresses some of the underlying statistical assumptions and issues in the collection and refinement of powder diffraction data. While standard data collection and Rietveld analysis have been extremely successful in providing structural information on a vast range of materials, there is often uncertainty about the true accuracy of the derived structural parameters. In this paper, we discuss a number of topics concerning data collection and the statistics of data analysis. We present a simple new function, the cumulative chi-squared distribution, for assessing regions of misfit in a diffraction pattern and introduce a matrix which relates the impact of individual points in a powder diffraction pattern with improvements in the estimated standard deviation of refined parameters. From an experimental viewpoint, we emphasise the importance of not over-counting at low-angles and the routine use of a variable counting scheme for data collection. Data analysis issues are discussed within the framework of maximum likelihood, which incorporates the current least-squares strategies but also enables the impact of systematic uncertainties in both observed and calculated data to be reduced.

## 1. Introduction

We can improve the quality of the structural results obtained from a powder diffraction pattern by a number of means. Firstly and most importantly, sufficient care should be taken in performing a good experiment and the observed diffraction data should be as free from systematic errors as possible. Due attention should be given to all parts of the diffraction pattern. The relative importance of, for example, low- and high-angle regions of a diffraction pattern should be assessed before performing the experiment and consideration paid to the balance of data collection statistics across the diffraction pattern. With structure solution and refinement from x-ray powder diffraction data, we stress the importance of a variable counting scheme that puts substantially increased weight on the high-angle reflections and explain why over-counting low-angle reflections can be deleterious to obtaining accurate structural parameters.

After determining the best data collection protocol, the next consideration for obtaining good quality structural results is ensuring that the calculated diffraction pattern is modelled well. For example, a good understanding of the profile line shape through a fundamental parameters technique pays dividends in obtaining a good fit to the Bragg peak shape.

On first thought, it might be expected that the combination of a careful experiment followed by careful modelling of the diffraction data is all that needs be considered to obtain good structural information. However, there is an important third facet that is rarely actively considered and indeed generally taken for granted—the algorithm behind fitting the model to the data. We generally assume that least-squares analysis is sufficient and indeed it is often so. However, least-squares is usually employed “because that’s the way it has always been done” rather than because of a positive consideration of its applicability. This mirrors the experimental situation mentioned earlier where constant-time data-collection approaches are still often preferred over variable counting-time strategies despite the fact that it has been known for at least a decade that the latter procedure gives better, more accurate results for x-ray powder diffraction data [1,2].

The underlying principles of probability theory indicate that least-squares analysis is appropriate only if (i) the data points have an associated Gaussian error distribution and (ii) the proposed model is a complete representation of the observed data. Although these conditions appear to be rather restrictive, they are nevertheless broadly satisfied in most Rietveld analyses. One exception to standard least-squares analysis that was discussed several years ago is the situation where the counts per data point are low (≤20) and followed a Poisson rather than a Gaussian distribution. Antoniadis et al. showed that a maximum likelihood refinement with due account given to Poisson counting statistics was the correct approach [3]. Indeed, maximum likelihood and Bayesian probability theory offer the correct formalism for considering all data and model uncertainties; least-squares analysis is just one, albeit relatively general, instance of maximum likelihood. Careful consideration of the physical origins of uncertainties in either data errors or insufficiencies in the structural model leads to probability distribution functions that must be optimised through maximum likelihood methods.

The fundamental statistics approach that looks for a physical understanding of the uncertainties in a powder diffraction pattern is in many ways analogous to the fundamental parameters approach used in peak shape analysis. Both methods of analysis lead to more reliable results. In this paper, several applications of maximum likelihood that go beyond least-squares analysis are discussed. These include dealing with unknown systematic errors in the data, unattributable impurity phases and incomplete structural model descriptions.

## 2. Assessing the Quality of a Rietveld Refinement

Before considering how we can optimise our chances of success using improved data collections methods or alternative statistical approaches, it is worth benchmarking the statistical quality of the Rietveld fit to a powder diffraction pattern. The conventional goodness-of-fit quantities used in the Rietveld method are the standard *R*-factors and *χ*^2^ quantities. The following four *R*-factors are generally quoted in most Rietveld refinement programs:expected *R*-factor:

$$\begin{array}{l} R_{E}=\sqrt{(N-P+C)/\left(\sum_{i=1}^{N}w_{i}y_{i}^{2}\right)} \end{array}$$ (1a)

weighted profile *R*-factor:

$$\begin{array}{l} R_{\text{wP}}=\sqrt{\left(\sum_{i=1}^{N}w_{i}{\left(y_{i}-M_{i}\right)}^{2}\right)/\left(\sum_{i=1}^{N}w_{i}y_{i}^{2}\right)} \end{array}$$ (1b)

profile *R*-factor:

$$R_{P}=\sqrt{\left(\sum_{i=1}^{N}{\left(y_{i}-M_{i}\right)}^{2}\right)/\left(\sum_{i=1}^{N}y_{i}^{2}\right)}$$ (1c)

Bragg *R*-factor:

$$R_{B}=\sqrt{\left(\sum_{k=1}^{N_{\text{ref}}}{\left(I_{k}^{\text{obs}}-I_{k}^{\text{calc}}\right)}^{2}\right)/\left(\sum_{i=1}^{N}{\left(I_{k}^{\text{obs}}\right)}^{2}\right)}$$ (1d)

The expected *R*-factor is basically as good as the weighted profile *R*-factor can get since the weighted sum of the squares of the difference between observed and calculated profile values, $\sum_{i=1}^{N}w_{i}{\left(y_{i}-M_{i}\right)}^{2}$, can at best be equal to the number of independent data, (*N*–*P*+*C*), in the diffraction pattern since each weighted squared profile difference in a best fit to the data should be equal to unity. In a standard x-ray powder diffraction pattern, the weight, *w_i_*, is equal to 1/*y_i_*. Since *N* is generally much larger that either *P* or *C*, then the expected profile *R*-factor can be rewritten as

$$\begin{array}{l} R_{E}=\sqrt{(N-P+C)/\left(\sum_{i=1}^{N}w_{i}y_{i}^{2}\right)}\approx \sqrt{N/\left(\sum_{i=1}^{N}y_{i}^{2}/y_{i}\right)}\\ \;\approx 1/\sqrt{\left(<y>\right)}. \end{array}$$ (2)

The expected profile *R*-factor is thus equal to the reciprocal of the square root of the average value of the profile points. A small expected profile *R*-factor is simply a statement about quantity and means that the average number of counts in a profile is large—it bears no relationship to the quality of a profile fit. In particular, if the diffraction pattern consists of weak peaks on top of a high background, then the expected *R*-factor can be very low. For an average background count of 10 000, for example, the expected *R*-factor will be 1 % or lower irrespective of the height of the Bragg peaks. This has led to a preference for quoting background-subtracted (b–s) *R*-factors,(b–s) expected *R*-factor:

$$R_{(\text{b-s})E}=\sqrt{(N-P+C)/\left(\sum_{i=1}^{N}w_{i}{\left(y_{i}-b_{i}\right)}^{2}\right)}$$ (3a)

(b–s) weighted profile *R*-factor:

$$R_{(\text{b-s})\text{wP}}=\sqrt{\left(\sum_{i=1}^{N}w_{i}{\left(y_{i}-M_{i}\right)}^{2}\right)/\left(\sum_{i=1}^{N}w_{i}{\left(y_{i}-b_{i}\right)}^{2}\right)}.$$ (3b)

The (b–s) expected *R*-factor gives a much more realistic measure of the quality of the data $\left(R_{(\text{b-s})E}\approx 1/\sqrt{\left[{\left(y-b\right)}^{2}/y\right]}\right)$ and the (b–s) weighted *R*-factor to both the quality of the data and the quality of the fit to the data. However, even still there are caveats. Very fine profile steps in a diffraction pattern lead to higher expected *R*-factors. For a given diffraction pattern, doubling the step size (i.e., grouping points together in pairs) will lead to an expected *R*-factor that is roughly $\sqrt{2}$ smaller than before. Additionally, *R*-factors may also be quoted for either the full profile or only those profile points that contribute to Bragg peaks. In themselves, therefore, profile *R*-factors treated individually are at best indicators of the quality of the data and the fit to the data. However, the ratio of weighted profile to expected profile *R*-factors is a good measure of how well the data are fitted. Indeed, the normalised *χ* ^2^ function is simply the square of the ratio of *R*_wp_ and *R*_exp_:

$$\begin{array}{l} \chi^{2}=\sum_{i=1}^{N}w_{i}{\left(y_{i}-M_{i}\right)}^{2}/(N-P+C)=\\ \;{\left(R_{\text{wP}}/R_{E}\right)}^{2}={\left(R_{(\text{b-s})\text{wP}}/R_{(\text{b-s})E}\right)}^{2} \end{array}$$ (4)

(Note that the *R*-factor ratio holds whether or not the background has been subtracted in the calculation of the *R*-factor. The *χ* ^2^ value will change, however, if only those points that contribute to Bragg peaks are considered instead of the full diffraction pattern.)

Bragg *R*-factors are quoted as an indicator of the quality of the fit between observed and calculated integrated intensities. It has been shown that the correct integrated intensity *R*-factor can be obtained from a Pawley or Le Bail analysis [4] where the extracted “clumped” integrated intensities, (*J_h_*) = ∑ (*I_h_*), are matched against the calculated “clumped” intensities, *J_h_* = ∑ *I_h_*, through the following equations:expected *R*_I_-factor:

$$R_{(I)E}=\sqrt{(N_{\text{clump}}-N_{x}+C_{x})/\left(\sum_{h=1}^{N_{\text{clump}}}\sum_{k=1}^{N_{\text{clump}}}W_{hk}(J_{h})(J_{k})\right)}$$ (5a)

*R*_I_-factor:

$$R_{\left(I\right)}=\sqrt{\left(\sum_{h=1}^{N_{\text{clump}}}\sum_{k=1}^{N_{\text{clump}}}W_{hk}[J_{h}-(J_{h})][J_{k}-(J_{k})]\right)/\left(\sum_{h=1}^{N_{\text{clump}}}\sum_{k=1}^{N_{\text{clump}}}W_{hk}(J_{h})(J_{k})\right)}$$ (5b)

where a “clump” is a group of completely overlapped reflections and the weight matrix *W_hk_* is the associated Hessian matrix from the Pawley analysis. It is easily shown that

$$W_{hk}=\sum_{i}w_{i}\;p(x_{i}-x_{h})p(x_{i}-x_{k})$$

where *p* (*x_i_–x_k_*) is the normalised peak shape for reflection *k* which is situated at *x_k_* These weights are calculated as part of the Pawley analysis but are easily calculated independently and therefore the above *R*-factors may also be derived from a Le Bail analysis. The integrated intensities *χ* ^2^ is again simply the square of the ratio of weighted and expected *R*-factors:

$$\begin{array}{l} \chi_{I}^{2}=\sum_{h=1}^{N_{\text{clump}}}\sum_{k=1}^{N_{\text{clump}}}W_{hk}[J_{h}-(J_{h})][J_{k}-(J_{k})]/(N_{\text{clump}}-N_{x}+C_{x})\\ \;={\left(R_{I}/R_{(I)E}\right)}^{2}. \end{array}$$ (6)

There is a strong argument that the estimated standard deviations of the structural parameters obtained from a Rietveld analysis should be multiplied by the square root of this *χ* ^2^ function rather than, as is conventional, the square root of the Rietveld *χ* ^2^. This usually leads to an additional inflation of between a factor of 2 and 4 for the estimate of the standard deviations of the structural parameters [4]. Interestingly, *χ*_I_^2^ can be evaluated indirectly from a combination of Rietveld and Pawley analyses on a dataset. Within statistical errors the numerator of the Rietveld *χ* ^2^ function (i.e., the unnormalised Rietveld *χ* ^2^ function) is equal to the sum of the unnormalised Pawley and integrated intensity *χ* ^2^ functions [4], i.e.,

$$\begin{array}{l} \;\sum_{i}w_{i}{\left(y_{i}-M_{i}^{R}\right)}^{2}≅\sum_{i}w_{i}{\left(y_{i}-M_{i}^{P}\right)}^{2}+\\ \sum_{h=1}^{N_{\text{clump}}}\sum_{k=1}^{N_{\text{clump}}}W_{hk}[J_{h}-(J_{h})][J_{k}-(J_{k})]. \end{array}$$ (7)

In this section, we have shown that there are a plethora of *R*-factors and *χ* ^2^ functions that may be used to evaluate the quality of and the quality of fit to a powder diffraction pattern. Probably the most useful set of quantities to use are the following:

- the background-subtracted, expected profile *R*-factors evaluated over (a) full profile and (b) Bragg peaks only (two quantities)
- the background-subtracted, weighted profile Rietveld and Pawley (or Le Bail) *R*-factors evaluated over (a) full profile and (b) Bragg peaks only (four quantities)
- the Rietveld and Pawley (or Le Bail) *χ* ^2^ functions evaluated over (a) full profile and (b) Bragg peaks only (two quantities)
- the expected and weighted integrated intensity *R*-factors and associated *χ* ^2^ (three quantities)

These quantities together give an indication of how well the profile data are fitted using (a) only the unit cell, peak shape and other profile parameters (Pawley/Le Bail quantities) and (b) a structural model (Rietveld quantities). The quantities associated with the integrated intensities allow a broad comparison to be made with single crystal results.

As a final point in the discussion of *R*-factors, it is worth noting that while expected Rietveld *R*-factors will always improve with additional counting time, *t*, (indeed it is straightforward to show from Eq. (2) that $R_{E}\propto 1\sqrt{t}$) the weighted profile *R*-factor bottoms out at a constant value that does not improve with time. This happens because the model cannot fit the data any better and it is systematic errors that are dominating the misfit. Indeed, David and Ibberson have shown that counting times are often an order of magnitude longer than necessary and that most datasets are probably over-counted—these conclusions corroborate earlier work by Baharie and Pawley [5,6].

## 3. The Cumulative *χ* ^2^ Distribution

**Fig. 1:**
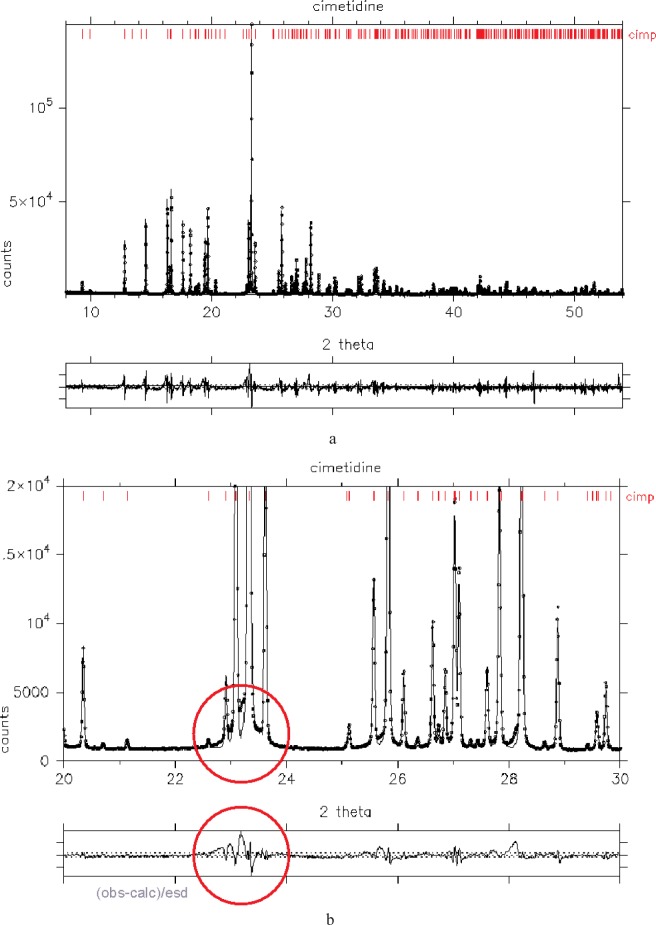

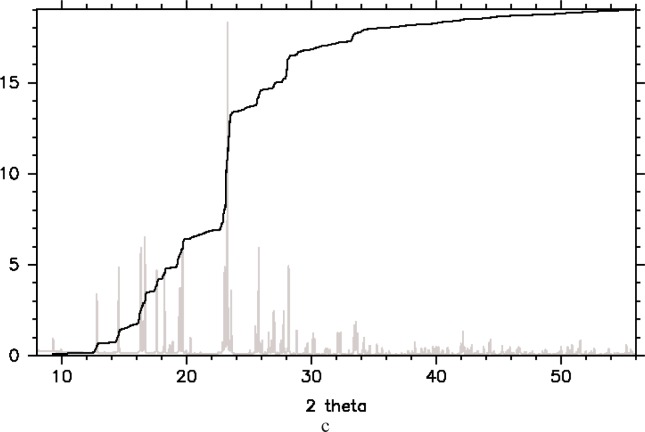
Observed and calculated diffraction pattern of cimetidine. Tick marks indicate the positions of Bragg peaks while the lower panel graph shows the difference/esd (the dotted lines represent ±3 *σ* (a) the full diffraction pattern (b) expanded range between 20° and 30° highlighting the region of major misfitting (c) the full diffraction pattern along with the cumulative chi-squared distribution.

In the previous section, we showed that the Rietveld *χ*^2^ function was a good measure of the quality of fit to a powder diffraction pattern. Examining, Eq. (4), it is clear that *χ*^2^ is the weighted sum of the squares of the difference between observed and calculated powder diffraction patterns. An auxiliary plot of the “difference/esd” underneath a fitted powder diffraction pattern gives a good idea of where the pattern is fitted well and where it is fitted poorly. Figure 1a shows the fitted diffraction pattern for cimetidine collected on station 2.3 at Daresbury. From the “difference/esd” plot, regions of misfit can clearly be seen around some of the strongest Bragg peaks between 22° and 24°. However, the “difference/esd” plot only gives a qualitative impression of how poor the fit is, even when the plot of the diffraction pattern is expanded (Fig. 1b). To assess the impact of a Bragg peak or a region of the diffraction pattern on the overall fit to the data, we need to assess the cumulative impact over that region. This can be achieved by plotting the cumulative chi-squared function which is the weighted sum of the squares of the difference between observed and calculated powder diffraction patterns up to that point in the diffraction pattern. The cumulative chi-squared function at the *n*th point in the diffraction pattern is given by

$$\chi_{n}^{2}=\sum_{i=1}^{n}w_{i}{\left(y_{i}-M_{i}\right)}^{2}/(N-P+C).$$ (8)

Examination of Fig. 1c shows that this function gives a clear indication of where the principal areas of misfit are in the powder diffraction pattern of cimetidine. The region from 22° and 24° is indeed the worst area of profile fit in the powder diffraction pattern. Around one third of the total *χ* ^2^ value is attributable to this small region. Moreover, the first half of the pattern contributes to ≈17/19 = 90 % of the total misfitting. The cumulative chi-squared plot clearly highlights the problems in fitting the cimetidine data and provides pointers to improving the fit to the data and hence obtaining an improved more accurate structural model. Indeed, there are three directions that we can take to improve the quality of profile fitting:

- redo the experiment to count for shorter times at low two-theta values and for longer at higher two-theta values. This will reduce the cumulative *χ* ^2^ contribution in the 22° and 24° region and up-weight the well-fitted high angle data (see Sec. 4.1).
- develop an improved model to describe the diffraction pattern—a good example of this might be the inclusion of anisotropic line broadening.
- downweight the regions of misfit if it proves difficult to obtain a simple model. (In the 22° and 24° region, the misfitting may occur as a consequence of disorder diffuse scattering—most codes do not include this effect.) In such cases, downweighting the misfitting points appropriately will lead to improved, less biased structural parameters (see Sec. 5.1 and Ref. [7]).

## 4. Assessing the Impact of Specific Regions of a Powder Diffraction Pattern

In the previous section, we discussed global measures of the quality of a Rietveld fit to a powder diffraction pattern. Ideally, we would like to be able to go further and devise an optimal methodology for collecting diffraction data. What parts of a powder diffraction pattern have the maximum impact on improving the quality of a crystal structure refinement? What parts of a diffraction pattern, for example, contribute most to the precise determination of anisotropic displacement parameters? The intuitive answer is that high angle reflections will be the most important but peak overlap will reduce this impact. In fact, both low and high angles regions (but, to a lesser extent, intermediate regions) are generally important. The counterintuitive importance of the low angle reflections results from the correlation of anisotropic displacement parameters with the scale factor. How does one then assess the impact of a single point in a diffraction pattern on the precision of a particular structural parameter? Prince and Nicholson showed for single crystal diffraction that the impact of individual reflections may be assessed statistically using standard least squares analysis [8]. Their procedure is easily extended to powder diffraction data [9].

The covariance matrix, ***V***, obtained from Rietveld analysis is the best measure of the precision and correlation of the refined parameters, *p_j_*, *j* = 1,…, *N*_par_, from a powder diffraction pattern containing *N*_obs_ points; *x_i_*, *y_i_* and *e_i_* are, respectively, the position profile value and estimated standard deviation of the *i*th point in the pattern which is modelled by a function value, *M_i_*. The covariance matrix, ***V***, is the inverse of the Hessian matrix, ***H***, which may be expressed as ***H*** = ***A*^T^*wA*** where the elements of ***A*** are *A_ij_* = ∂*M_i_* / ∂*p_j_* and ***w*** is the weight matrix which is usually diagonal with elements $w_{ii}=1/\sigma_{i}^{2}$. Forming the matrix ***Z*** with elements *Z_ij_* = (1/*σ_i_*) ∂*M_i_* / ∂*p_j_* means that the Hessian matrix may be written as ***H*** = ***Z*^T^*Z***. From this ***Z*** matrix, the projection matrix, ***P***, may be formed from the equation ***P*** = ***Z***(***Z*^T^*Z***)^−1^***Z*^T^** [8]. This matrix, although not often discussed in least squares analysis, has a number of important properties. Most notably, the on-diagonal element, *P_ii_*, is the leverage of a data point and has a value between zero and one. A high leverage means that a data point plays an important role in the overall model fitting and vice-versa. There is, however, another significant quantity for the analysis of the variance of a particular parameter.

Consider the impact on a particular element *V_rs_* of the covariance matrix if the *i*th data point is collected for a fraction *α_i_* longer. The Hessian matrix is modified as follows: ***H***′ = ***H*** + *α _i_****z*^T^*z*** where the row vector ***z*** has elements *z_j_* = (1/*σ_i_*) ∂*M_i_* / ∂*p_j_*. Since the Hessian and covariance matrices are the inverses of each another, the change in the covariance matrix may be shown to be

$$V^{\prime }=V-\alpha_{i}(Vz^{T}zV)/(1+\alpha_{i}z^{T}\mathrm{Vz})$$ (9)

This equation may be simplified when it is recognised that ***z*^T^*Vz*** = *P_ii_*. Putting the vector ***t*** = ***zV*** implies that (***Vz*^T^*zV***)_rs_ = (***zV***)**^T^**_r_(***zV***)_s_ = *t*_r_*t*_s_ and thus, as long as *α* is small, all the elements of the parameter covariance matrix are altered as follows:

$${V^{\prime }}_{\text{rs}}=V_{\text{rs}}-\alpha_{i}(t_{r}t_{s})/(1+\alpha_{i}P_{ii})≅V_{\text{rs}}-\alpha_{i}t_{r}t_{s}.$$ (10)

The product *t*_r_*t*_s_ is thus a measure of the impact of the *i*th point on element *rs* of the covariance matrix. In particular, $t_{j}^{2}$ is a measure of the importance of the *i*th data point on the *j*th parameter; a large value of $t_{j}^{2}$ leads to a substantial reduction in the parameter variance and a concomitant improvement in precision. The quantity

$$t_{r}(i)=\sum_{s=1}^{N_{\text{par}}}\frac{1}{\sigma_{i}}\frac{\partial M_{i}}{\partial p_{s}}V_{\text{rs}}$$ (11)

is perhaps more informative than its square as it provides information about the sense of the *i*th data point contribution to the covariance terms. Its relationship to the covariance matrix is essentially identical to the relationship between the residual (observed-calculated)/(estimated standard deviation) and the overall *χ* ^2^ goodness of fit. A specific example^1^ of the use of the *t*-matrix to determine the significance of different parts of a powder diffraction is discussed in Ref. [9].

### 4.1 Variable Counting Time Protocols for X-Ray Powder Diffraction Data Collection

The use of *t*_r_(*i*) as a diagnostic for determining accurate structural parameters depends on whether we believe that the errors in our data are well understood or not. If we are sure that the sources of the errors in our data are all known—the simplest case is the belief that the only sources of uncertainty are from counting statistics—then we will target those points in the diffraction pattern that have the maximum values of *t*_r_(*i*) since these will be the points that reduce the estimated standard deviations of a parameter by the greatest amount. It is intuitively obvious that we will get the most precise assessment of the area of a peak by counting for longest at the top of the peak and that we will get the best indication of the peak position by counting at the points of maximum gradient change on the peak. These conclusions, however, do depend on us knowing with complete confidence what the peak shape is. This point, in turn, means that we can only use these maximum impact points if we not only know that source of all our experimental errors but also have complete confidence in our model. While this may often be true for neutron powder diffraction data, it is generally not the case for x-ray diffraction and patterns such as those shown for cimetidine in Fig. 1 are the norm rather than the exception. If we were entirely confident about the sources of misfit in our low-angle diffraction data then we would count for longer at low angles since this offers the prospects of reducing the terms in the covariance matrix by the largest amount. If we are uncertain about our data errors and the sufficiency of our model then we have to take an alternative approach to the problem that is effectively opposite to the argument when the errors are known. If we have an intense Bragg peak at low angles and are uncertain about our errors then *t*_r_(*i*) tells us that the variance terms will reduce substantially but unfortunately in an incorrect way. We will have a more precise result but a less accurate one. Indeed, as the variance terms reduce, we will be faced with a result that may be increasingly more precise while at the same time decreasingly accurate. To obtain accurate results in the face of uncertain errors, our best approach is to distribute the errors as evenly as possible across all the Bragg peaks. This means counting for substantially longer at higher angles. There are two published methods for deciding how to vary the counting time across the diffraction pattern [1,4,10]. Both approaches lead to essentially identical protocols and also both lead to the important conclusion that higher angle parts of the diffraction pattern may have to counted for often more than 30 times longer than low-angle regions. In order to explain the rationale for longer counting times, we follow the approach of David [4] and Shankland, David and Sivia [10] that was developed with a view to improving the chances of structure solution. The rationale is based upon one of the central formulae of Direct methods, the tangent formula which determines the probable relationship between the phases, *ϕ* (***h***), *ϕ* (***k***) and *ϕ* (***h***–***k***):

$$\tan[\varphi (h)]≅\frac{\sum_{k}\frac{\sigma_{3}}{\sigma_{2}^{3/2}}E(h)E(k)E(h-k)\sin[\varphi (k)+\varphi (h-k)]}{\sum_{k}\frac{\sigma_{3}}{\sigma_{2}^{3/2}}E(h)E(k)E(h-k)\cos[\varphi (k)+\varphi (h-k)]}$$ (12)

where $\sigma_{n}=\sum_{i-1}^{n}{\left[f_{i}(\left|h\right|=0)\right]}^{n}$ and the normalised structure factor, *E*(***h***), is related to the integrated intensity, *I* [(***h***)] = *j*(***h***) |*F*(***h***)^2^| by the equation ${\left|E(h)\right|}^{2}=I(h)/\sum_{j=1}^{n}g_{j}^{2}(h)$.^2^ We simply require that the fractional error in *E*(***h***) should be independent of where the reflection is in the diffraction pattern. This, in turn, leads to the fact that all components of the summations in the tangent formulae will on average be determined with equal precision. When we collect a powder diffraction pattern, the Bragg peak area, *A*(***h***), is not the integrated intensity itself but is modified by geometrical, absorption and extinction terms. If we know that absorption and extinction effects are severe, then we should include their effects in evaluating the variable collection strategy. However, if we work under the simpler assumption that these effects are small, then *A* (***h***) = *L*_p_*I* (***h***), where *L*_p_ is the Lorentz polarisation correction and we will count normalised structure factors, *E* (***h***), with equal precision across a powder diffraction pattern if we offset the combined effects of *L*_p_, the form-factor fall-off and the Debye-Waller effects of thermal motion, i.e., $t(2\theta )\propto 1/L_{p}(2\theta )\sum g_{j}^{2}(2\theta )$ where we have explicitly used a 2-theta dependence. For the case of Bragg-Brentano geometry on a laboratory-based x-ray powder diffractometer, this becomes

$$t(\theta )\propto \frac{\left(\sin\theta \sin2\theta )(1+\cos^{2}2\alpha \right)}{\left(1+\cos^{2}2\alpha \;\cos^{2}2\theta )f_{\text{av}}^{2}(\theta )\exp(-2B_{\text{av}}\sin^{2}\theta /\lambda^{2}\right)}$$ (13a)

where *f*_av_ is a representative atomic scattering factor (e.g., carbon), *B*_av_ is an estimated overall Debye-Waller factor, *λ* is the incident wavelength and 2*α* is the mono-chromator take-off angle. For the case of Debye-Scherrer geometry on a synchrotron x-ray powder diffractometer, this simplifies to

$$t\theta \propto (\sin\theta \;\sin\theta )/[f_{\text{av}}^{2}(\theta )\exp(-2B_{\text{av}}\sin^{2}\theta /\lambda^{2})].$$ (13b)

**Fig. 2:**
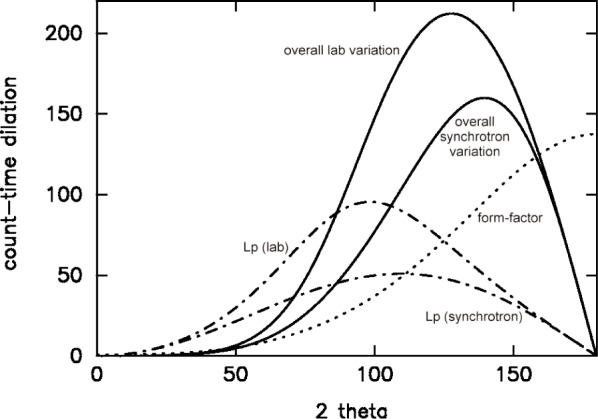
Variable counting time schemes for both laboratory and synchrotron diffractometers. The dilation is normalised to be unity for 2*θ* = 10°.

**Fig. 3:**
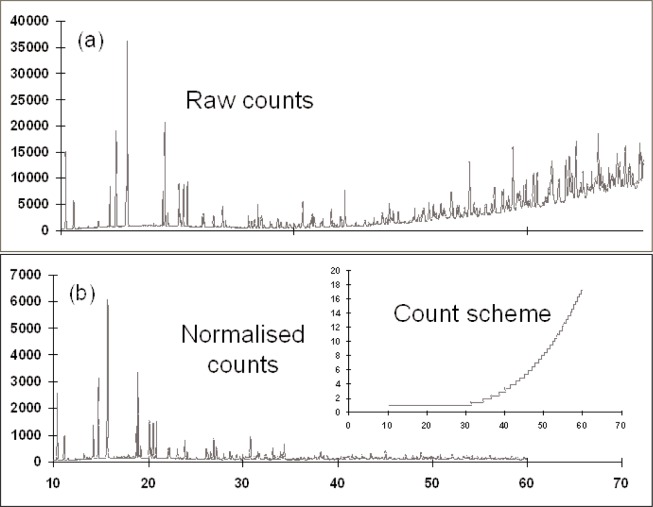
Raw and normalised counts for synchrotron powder diffraction data of chlorothiazide. The inset shows the variable counting scheme used.

**Fig. 4:**
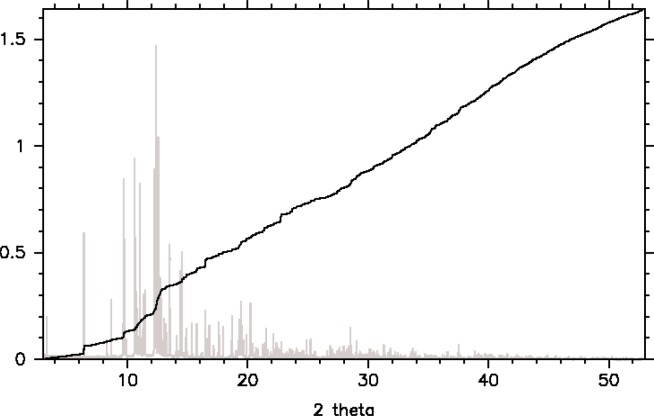
The cumulative chi-squared distribution for famotidine overlaid upon the synchrotron powder diffraction pattern. The benefits of the variable counting time scheme are clear as the impact of all regions of the pattern are similar.

The variable counting time scheme for these two typical diffractometer settings are shown in Fig. 2. Both laboratory and synchrotron variations show that the counting times at intermediate angles should be substantially longer than at low-angles and extreme backscattering. Interestingly, the 2-theta variations of the variable counting time schemes are dominated as much by the Lorentz polarisation correction as the form-factor fall-off and Debye-Waller variation. Indeed at low-angles, the principal effects are associated with the Lorentz polarisation correction. All three effects combine together to create a substantial variation in counting time as a function of 2-theta. Figure 3 compares the constant counting time pattern (Fig. 3a) compared with the raw counts using the variable counting time protocol (Fig. 3b) for the drug compound, chlorothiazide. The Bragg peaks at high angle appear to be of the same intensity as the low-angle reflections —all the Bragg peaks in this diffraction pattern have been reliably determined. This proved crucial in the successful structure solution of the compound using Direct methods as large numbers of reliable triplet phase relationships could be formed [10]. A further indication of the importance of using a variable counting time scheme can be seen from the analysis of the cumulative chi-squared distribution for the refinement of the structure of famotidine (Figure 4). The overall chi-squared is low (~1.6) showing that a good fit has been achieved over the full diffraction pattern. Moreover, the cumulative chi-squared distribution forms an essentially straight line over the full pattern indicating that all regions are fitted equally well and, as a corollary, that the errors are also even distributed over all the reflections. This is an important point as it follows from this that the effects of systematic errors must be substantially diminished over, for example, the case of cimetidine (see Fig. 1c).

## 5. Beyond Least-Squares Analysis

In the previous sections, we discussed from a statistical point of view how to assess the limitations of a Rietveld analysis and overcome these problems through the use of, for example, variable counting time protocols. What happens when we still have areas of the diffraction pattern that are not fitted well despite performing a careful experiment? If the misfit results from additional scattering from an unattributed impurity phase then we can formulate this within the context of Bayesian probability theory and develop an appropriate refinement procedure. If we have no real idea what has caused the misfitting—it may, for example, be lineshape effects, imperfect powder statistics or diffuse scattering—then we have to develop a catch-all probabilistic procedure for addressing this problem. If the misfitting involves a small proportion of the data, then we can develop a robust method of improving the accuracy of our results. At the same time, however, our precision decreases because we have allowed the possibility of more sources of uncertainty than in a standard least-squares analysis. The approach used in this paper follows that of Sivia who aptly discussed the problem as one of “dealing with duff data” [11].

### 5.1 Dealing With Duff Data

When we observe misfitting in a powder diffraction pattern, our first assumption is that the structural model that we have used to describe the data is not quite optimised. However, we often find that despite our best attempts, the data never fit well across the full diffraction pattern and we are left with regions of misfit that may well be introducing systematic errors into our data. If we understand the source of this misfit—it may for example be an unattributable impurity phase—then we may be able to develop a suitably specific maximum likelihood refinement protocol. However, when we are unable to postulate a suitable explanation for misfitting, then we must develop a very general probabilistic approach, as has been done previously [11,12]. If we take a standard point in our diffraction pattern that has, say, 400 counts we know from Gaussian counting statistics that our expected standard deviation will be around 20 counts. If we proceed through to the end of our least squares analysis with this assumption, then we are making a very definite statement about our errors. We are saying categorically that we know all the sources of our errors and that they results only from counting statistics. Put in these terms, this is a bold assertion. Fortunately, in most Rietveld analyses (and particularly in the area of neutron powder diffraction) this is a fair statement to make. However, we will show that even with good refinements, we can improve our accuracy (at the expense of some precision) by using a more robust algorithm.

**Fig. 5:**
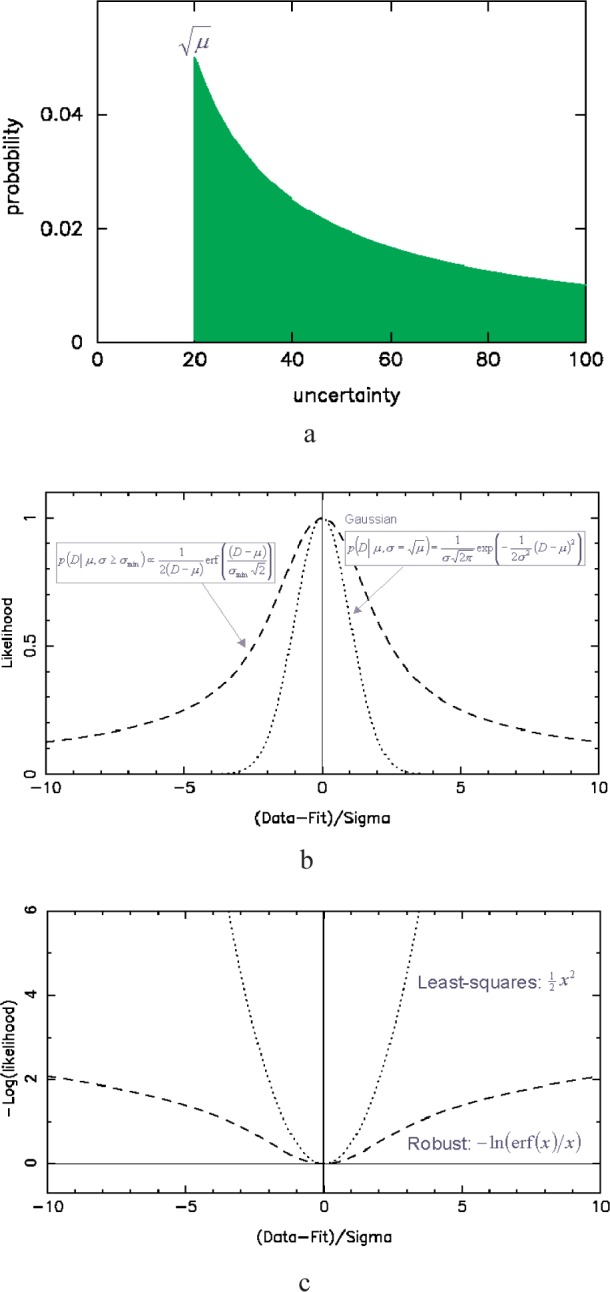
Robust least squares. (a) the probability distribution function associated with using the counting statistics error as a lower uncertainty bound and a scale-invariant Jeffrey’s prior to represent the degree of ignorance of other errors, (b) the standard least-squares likelihood (dotted line) compared with the robust likelihood (dashed line) derived from the probability distribution function shown in Fig. 5a, (c) the negative log-likelihood (or chi-squared equivalent) for standard least-squares (dotted line) and robust statistics (dashed line).

One of the things that we can say for sure when we have collected a point in our diffraction pattern with *μ* = 400 counts is that the uncertainty in our measurement cannot be less than 20 counts—but it could be more. We must generate a probability distribution for our uncertainty—after all, we are no longer certain about our uncertainties. A good distribution, because it has the properties of scale invariance, is the Jeffrey’s distribution, 1/*σ*, for all values $\sigma \geq \sqrt{\mu }$. This probability distribution for our uncertainty is shown in Fig. 5a. The corresponding likelihood for the data is obtained by integrating over this distribution

$$\begin{array}{l} p(D|\mu ,\sigma \geq \sqrt{\mu })=\\ \int_{\sigma_{\min}=\sqrt{\mu }}^{\infty }prob(\sigma )\frac{1}{\sigma \sqrt{2\pi }}\exp\left[-\frac{1}{2\sigma^{2}}{\left(D-\mu \right)}^{2}\right]d\sigma \end{array}$$ (14)

which leads, not to a Gaussian likelihood but an error-function distribution

$$p(D|\mu ,\sigma \geq \sigma_{\min})\propto \frac{1}{2(D-\mu )}erf\left[\frac{\left(D-\mu \right)}{\sigma_{\min}\sqrt{2}}\right].$$ (15)

This is shown in Fig. 5b. The negative log-likelihood, which gives a direct comparison with the least-squares distribution, is shown in Fig. 5c. For large positive and negative deviations between observed and calculated data, the penalty no longer follows a quadratic form but rather a logarithmic distribution. Large deviations have less impact on this robust modified *χ* ^2^ function while small deviations are treated just like the standard least-squares (albeit with a shallower distribution arising from our poorer state of knowledge about our uncertainties).

**Fig. 6:**
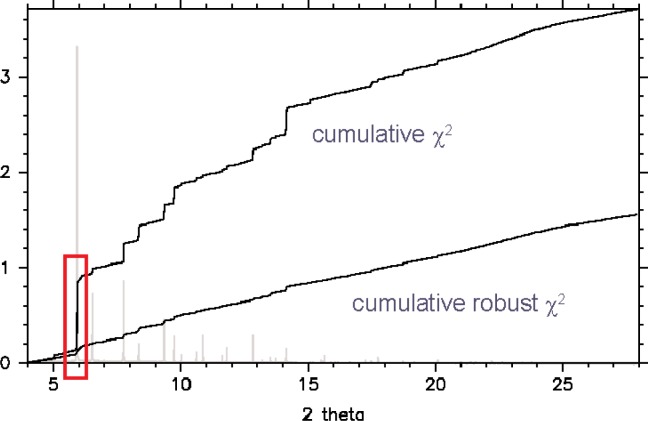
Comparison of the cumulative standard chi-squared function with the cumulative robust chi-squared function for urea. The synchrotron powder diffraction pattern of urea is shown in the background.

We illustrate the use of this robust statistic for the case of a high resolution x-ray powder diffraction pattern of urea collected on BM16 at the ESRF, Grenoble. Standard least-squares analysis leads to a satisfactory weighted profile *χ* ^2^ of ~3.7. However, examination of the cumulative *χ* ^2^ plot (Fig. 6), shows that almost a quarter of the misfit is associated with the strongest Bragg peak. This could result from preferred orientation, detector saturation or particle statistics—we don’t know. The cumulative robust *χ* ^2^ distribution, on the other hand, contains no such bias towards this single peak. Indeed, the linear variation of the cumulative robust *χ* ^2^ distribution across the full pattern gives a reassuring degree of confidence to this modified least-squares approach. However, a comparison of the structural parameters for the conventional and robust least-squares approaches with single crystal data convincingly shows the benefits of the robust metric which automatically downweights bad data. With conventional least-squares, the results are good and the estimated standard deviations are small. However, nine of the fourteen structural parameters are more than four standard deviations different from their single crystal counterparts indicating that the accuracy of the parameters obtained from the least squares analysis does not measure up to their precision. On the other hand, only one of the structural parameters from the robust analysis is more than 4 *σ* away from the corresponding single crystal value. The parameters changes are modest between least-squares and robust analyses. However, the differences are real and the improvements in precision when benchmarked against the single crystal parameters are significant. While it is dangerous to extrapolate from a single example, the underlying statistical framework is sound and suggests that, when significant jumps are found in a cumulative chi-squared plot, then a robust analysis is worthwhile.

### 5.2 Refinement in the Presence of Unattributable Impurity Phases

What do you do when you want to perform a Rietveld analysis of a particular material but have a substantial impurity phase and despite all your best attempts you can neither remove it from your sample nor index it from your diffraction pattern? Conventional wisdom would state that your chances of obtaining unbiased structural parameters are poor and that the best you can do is to manually exclude the offending impurity peaks. Standard Rietveld programs that are based upon a least-squares refinement algorithm cannot cope in an unbiased manner with an incomplete model description of the data. This is just the situation where Bayesian probability theory can come to the rescue. We can ask the question, “How do I perform a refinement on a powder diffraction pattern when I know that there is an impurity phase present but have no idea what that impurity phase may be?” This question is equivalent to stating that my diffraction pattern contains a component that I can model (known phases + background) and an additional positive, unknown contribution. It turns out that enforcing the positivity of the unknown component as an additive contribution is sufficient to produce excellent results [7].

**Fig. 7:**
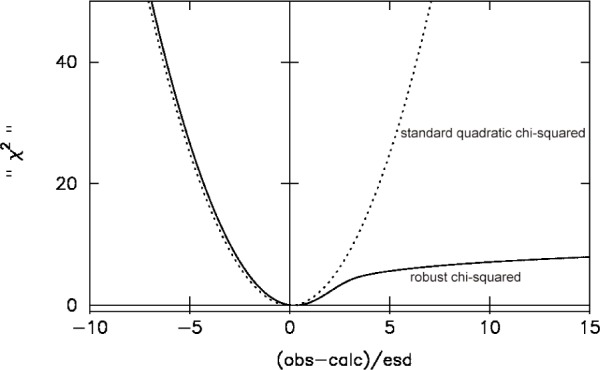
The modified robust goodness of fit function (solid line) compared with the standard quadratic least-squares function.

The mathematical development of these ideas has been presented elsewhere and results in a modified *χ* ^2^ goodness of fit function that is shown in Fig. 7 [7,13]. For observed data that are less than the model function, the new goodness of fit behaves essentially identically to the standard *χ* ^2^. This is to be expected since such points are unlikely to be associated with an impurity contribution. On the other hand, when the observed data value is substantially greater than the fitted model value, then the new goodness of fit brings a substantially smaller penalty (the function varies logarithmically) than the quadratic behaviour of the standard *χ* ^2^. Again this is just what is required to minimise the impact of any impurity phase. Note also that the curvature of the new goodness of fit is shallower than the standard *χ*^2^. This means that quoted standard deviations will be higher for refinements using the new goodness of fit. This is to be expected as the allowance for an impurity phase brings a greater uncertainty into the model parameter values.

**Fig. 8:**
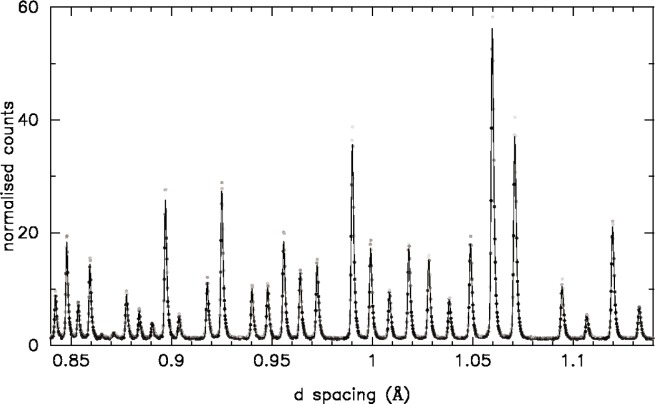
The observed and calculated diffraction patterns for pure yttria determined on HRPD at ISIS.

**Fig. 9:**
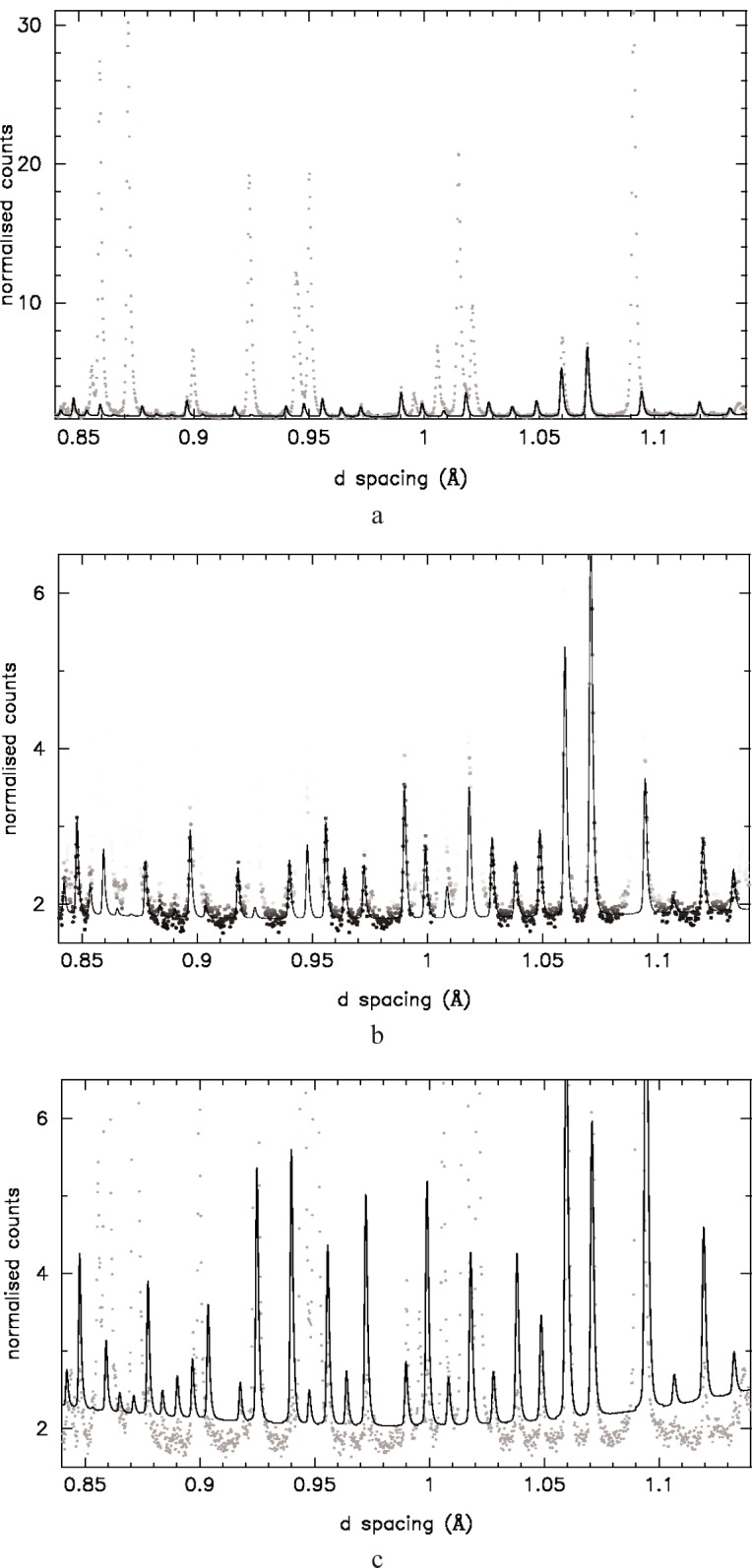
Observed and calculated diffraction patterns for the composition 5 % yttria : 95 % rutile: (a) robust analysis showing the full observed data range (the grey scale described in the text not used in this figure); (b) expanded region highlighting the successful robust refinement (the down-weighting grey scale is used in this figure); (c) the least-squares analysis showing the poor agreement between the observed and calculated patterns.

**Fig. 10:**
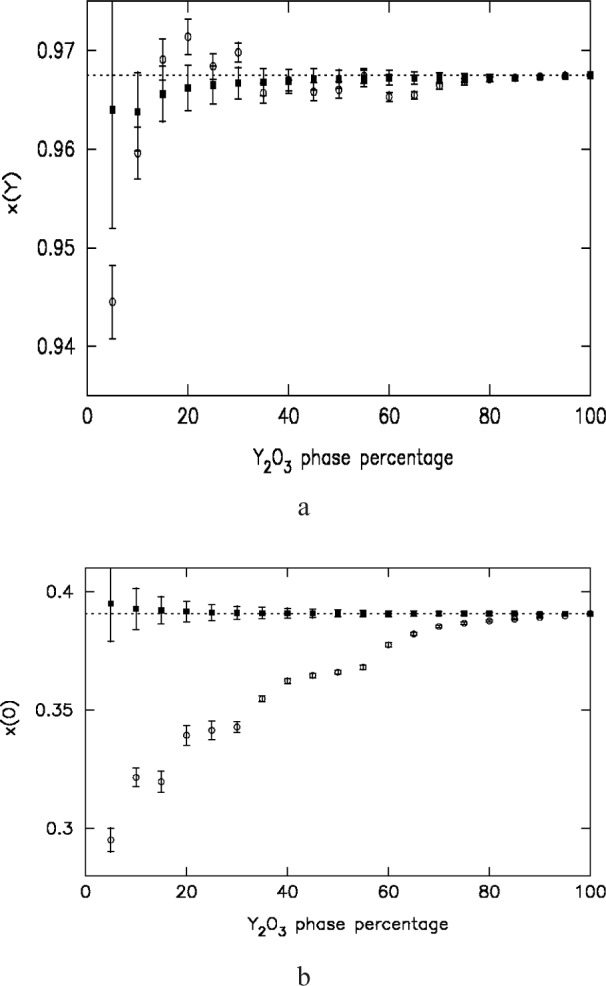

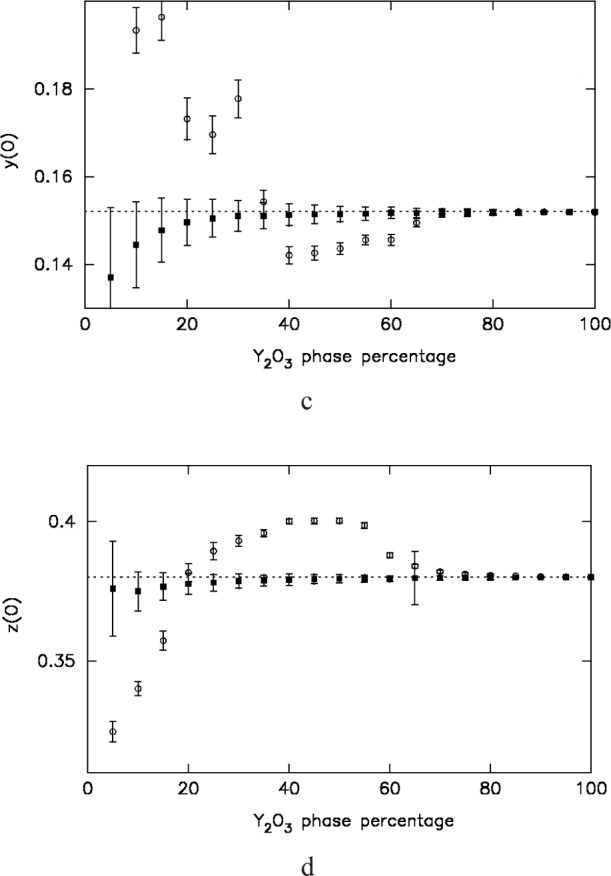
The refined atomic coordinates of yttria plotted as a function of yttria composition. Open circles and filled squares correspond to the least-squares and robust analyses, respectively. (a) The yttrium *x* coordinate. (b), (c), (d) The oxygen *x*, *y*, and *z* coordinates. The dotted lines correspond to the correct values obtained from least-squares refinement of the pure-yttria diffraction pattern.

Diffraction patterns of yttria and rutile were collected on HRPD at ISIS. Results from the 5 % yttria: 95 % rutile are shown in Fig. 9. (The fitted diffraction pattern of pure yttria is shown in Fig. 8 for comparison.) In order to accentuate the difference between the new goodness of fit function and standard least-squares analysis, we have chosen to refine the minority yttria phase treating the majority phase as the impurity (see Fig. 9a). The excellent fit to the data for the modified *χ*^2^ is shown in Fig. 9b where we have graphically down-weighted the observed points, which contribute least to the goodness of fit. This emphasises what the algorithm is effectively doing—large positive (obs-calc)/esd values are essentially ignored. In effect, the algorithm is optimally excluding those regions that do not contribute to the model. The relative calculated peak intensities agree very well with the results for pure yttria (Fig. 8). Least squares analysis (Fig. 9c) produces a completely different result—all points are considered with no downweighting for possible impurities. The first obvious effect is that the refined background is too high. The reason for this is obvious since the strong impurity peaks lift up the model fit. The relative peak intensities are however also very different from the correct values suggesting that the refined structural parameters are substantially in error. This is indeed the case and is borne out by analysis of the refined zirconium and oxygen coordinates, which are shown graphically in Fig. 10 as a function of yttia content. We briefly consider the other refined parameters (a fuller analysis is given in Ref. [7]). The scale factor is correct within estimated standard deviation (esd) for the robust analysis but behaves wildly for the standard least squares, exceeding 1000 % for 25 % yttria content. The least-squares analysis of the lattice constant also becomes increasingly unreliable as the refinement locks into peaks associated with rutile as well as yttria. On the other hand, the lattice constant from the robust refinement is satisfyingly stable; the esds increase as the yttria content decreases (the 5 % esd is some five times larger than the 100 % value) but all results lie within a standard deviation of the correct result.

### 5.3 Summary of Maximum Likelihood Refinement Algorithms

Least-squares Rietveld analysis is the best and least-biased method of structure refinement from a powder diffraction pattern when the data can be fully modelled. However, when there is an unmodelled contribution in the diffraction pattern, least-squares analysis gives biased results. In the impurity phase example discussed in this contribution, significant deviations from the correct parameter values occur when there is as little as a 10 % impurity contribution. At higher impurity levels, least-squares analysis is completely unreliable. These problems may, however, be overcome if the existence of an unknown impurity contribution is built into the refinement algorithm. While it might seem to be a logical inconsistency to build in information about an unknown contribution, Bayesian probability theory provides a framework for doing just this. Only two broad assumptions are necessary to derive an appropriate modified probability distribution function. These are (i) that the impurity contribution must be intrinsically positive and (ii) that its magnitude, *A*, is unknown and thus best modelled by a Jeffreys’ prior, given by *p*(*A* | *I*) ∝ 1/*A* for *A* > 0 and *p*(*A* | *I*) = 0 for *A* ≤ 0. This produces a modified “*χ* ^2^” function (see Fig. 1) that effectively excludes the impact of impurity peaks.

The results discussed in briefly in this contribution and more extensively in Ref. [13], show that the improvement over conventional least-squares analysis is dramatic. Indeed, even in the presence of very substantial impurity contributions (see Fig. 4) the refined structural parameters are within a standard deviation of their correct values.

It must, however, be stated as a final caveat that care should be taken with this approach and the use of an algorithm that can cope with the presence of impurities should be seen as a last resort. Indeed, every effort should be made to determine all the phases in a sample. It is much more desirable to include the impurity phase in a standard Rietveld refinement.

## Figures and Tables

**Table 1:** Structural parameters obtained for urea from single crystal results (column 2) and high-resolution powder diffraction data. Two separate analyses were performed on the powder diffraction data. Results from a standard least-squares analysis are shown in column 2 and compared with the single crystal results in column 3. The results from the robust analysis are listed in column 5 and compared with the single crystal results in the final sixth column. The shaded cells indicate discrepancies that are beyond 4 *σ*

	SXXD	Least squares	LS-SXXD	Robust	R-SXXD
C1 z	0.3328(3)	0.3236(9)	−0.0092(10)	0.3319(13)	−0.0009(14)
O1 z	0.5976(4)	0.6013(5)	0.0037(6)	0.5984(7)	0.0008(8)
N1 x	0.1418(2)	0.1405(3)	−0.0013(4)	0.1423(7)	0.0005(7)
z	0.1830(2)	0.1807(5)	−0.0023(6)	0.1813(7)	−0.0017(7)
C1 U_11_	0.0353(6)	0.0348(20)	−0.0005(20)	0.0329(40)	0.0024(40)
U_33_	0.0155(5)	0.0396(30)	0.0241(30)	0.0413(40)	0.0258(40)
U_12_	0.0006(9)	0.0205(30)	0.0199(30)	0.0128(40)	0.0122(40)
O1 U_11_	0.0506(9)	0.0749(16)	0.0243(18)	0.0617(30)	0.0111(30)
U_33_	0.0160(6)	0.0080(14)	−0.0080(15)	0.0090(20)	−0.0070(20)
U_12_	0.0038(18)	0.0052(20)	0.0014(30)	−0.0011(35)	−0.0049(35)
N1 U_11_	0.0692(6)	0.0627(15)	−0.0065(18)	0.0697(25)	0.0005(25)
U_33_	0.0251(4)	0.0460(22)	0.0211(22)	0.0365(30)	0.0114(30)
U_12_	−0.0353(7)	−0.0252(18)	0.0101(20)	−0.0361(30)	−0.0008(30)
U_13_	−0.0003(3)	−0.0015(11)	−0.0012(12)	−0.0029(15)	−0.0026(15)

## Footnotes

1 This example concerns the analysis of orientational order in C_60_ from neutron powder diffraction data. The *t*-matrix is used to show that the deviations from spherical symmetry of the orientation distribution function of C_60_ in the high temperature phase can be well modelled using neutron powder diffraction data and that powder averaging is quite different from spherical averaging.

2
$F(h)=\sum_{j=1}^{n}g_{j}(h)\exp(2\pi ih\cdot r)\;and\;g_{j}(h)=f_{j}(h)\exp(-B_{\text{av}}/4d^{2})$.
